# Supplementary material for: MetaRibo-Seq measures translation in microbiomes
Source: Nat Commun. 2020 Jun 29;11:3268. doi: 10.1038/s41467-020-17081-z (PMC7324362; doi:10.1038/s41467-020-17081-z)
Supplement: Supplementary file 10 — Supplementary Data 7 [file 41467_2020_17081_MOESM10_ESM.zip › File2/Confidence_High_Taxonomy/57473_out.krona.html]

Javascript must be enabled to view this page.

members
magnitude
magnitudeUnassigned
count
unassigned
taxon
rank

57473\_out

47

superkingdom
2
47

phylum
976
47

class
200643
47

47
171549
order

family
1
815

816
1
genus

species
1
357276

SRS053356\_contig\_number\_47034

family
171552
46

2
838
genus
46

SRS077641\_contig\_number\_4268SRS098585\_contig\_number\_contig-100\_2888.2888

44
165179

SRS011134\_contig\_number\_1183SRS013521\_contig\_number\_8416SRS013638\_contig\_number\_contig-100\_824.108568SRS013687\_contig\_number\_25653SRS014415\_contig\_number\_contig-100\_105.26958SRS015590\_contig\_number\_17179SRS015782\_contig\_number\_contig-100\_371.254887SRS015794\_contig\_number\_8037SRS016541\_contig\_number\_12366SRS017307\_contig\_number\_29395SRS017375\_contig\_number\_178SRS018888\_contig\_number\_7952SRS019285\_contig\_number\_contig-100\_323.324SRS022609\_contig\_number\_42707SRS023914\_contig\_number\_26545SRS024132\_contig\_number\_48805SRS043841\_contig\_number\_11209SRS044535\_contig\_number\_21520SRS045713\_contig\_number\_17026SRS049959\_contig\_number\_16044SRS049995\_contig\_number\_31713SRS050752\_contig\_number\_20400SRS053398\_contig\_number\_22743SRS054059\_contig\_number\_4194SRS058145\_contig\_number\_contig-100\_303.34906SRS064757\_contig\_number\_4651SRS065397\_contig\_number\_contig-100\_957.82684SRS074586\_contig\_number\_2823SRS077991\_contig\_number\_contig-100\_198.199SRS078176\_contig\_number\_11786SRS078419\_contig\_number\_6355SRS097958\_contig\_number\_18782SRS100907\_contig\_number\_contig-100\_6849.48906SRS103987\_contig\_number\_387SRS1041031\_contig\_number\_29177SRS1041140\_contig\_number\_1483SRS143181\_contig\_number\_1076SRS144297\_contig\_number\_3633SRS144506\_contig\_number\_52924SRS144537\_contig\_number\_36264SRS144603\_contig\_number\_13033SRS144714\_contig\_number\_199SRS147614\_contig\_number\_8374SRS148253\_contig\_number\_10701
species
